# Spatiotemporal dynamics of questing activity by four tick species in the central Great Plains

**DOI:** 10.1371/journal.pone.0351160

**Published:** 2026-09-01

**Authors:** Marlon E. Cobos, Joanna L. Corimanya, Eric Ng’eno, Claudia Nuñez-Penichet, Abigail C. Perkins, Zenia Ruiz-Utrilla, Abdelghafar Alkishe, Daniel Romero-Alvarez, Yuan Yao, Anuradha Ghosh, Xiangming Xiao, A. Townsend Peterson, Kathryn T. Duncan

**Affiliations:** 1 Department of Ecology and Evolutionary Biology & Biodiversity Institute, University of Kansas, Lawrence, Kansas, United States of America; 2 Fish and Wildlife Conservation Department, Virginia Tech, Blacksburg, Virginia, United States of America; 3 Research Group of Emerging and Neglected Diseases, Ecoepidemiology and Biodiversity, Health Science Faculty, School of Biomedical Sciences, Universidad Internacional SEK (UISEK), Quito, Ecuador; 4 School of Biological Sciences, Center for Earth Observation and Modeling, University of Oklahoma, Norman, Oklahoma, United States of America; 5 Biology Department, Pittsburg State University, Pittsburg, Kansas, United States of America; 6 Department of Veterinary Pathobiology, College of Veterinary Medicine, Oklahoma State University, Stillwater, Oklahoma, United States of America; University of North Dakota School of Medicine and Health Sciences, UNITED STATES OF AMERICA

## Abstract

The distributional expansions of several medically relevant tick species across the central Great Plains create a complex and shifting landscape of tick-borne disease risk. Traditional modeling approaches provide a general picture of distributional potential, but predictions are often static representations of species’ ranges. As pathogen transmission is directly linked to tick questing activity, characterizing the spatiotemporal heterogeneity of this behavior is essential to a better understanding of transmission risk and planning of interventions in the area. In this study, we explore novel ecological niche modeling frameworks based on full use of presence-absence data to illuminate the spatiotemporal dynamics of questing activity of four tick species in the central Great Plains region. We processed data from two years of field tick surveillance to derive presence and absence records of active ticks, maximizing sample sizes and making model evaluation more robust. We coupled sampling events with time-specific environmental covariates to leverage longitudinal sampling data still more. We also constrained model response curves to align with fundamental niche theory within a strong conceptual framework. Model outcomes demonstrate both seasonal dynamics of individual species and differences between species in terms of the geographic potential of questing behavior by Great Plains tick species. These modeling approaches provide a more robust basis for understanding ecological drivers of tick questing activity and characterizing the shifting landscape of vector-borne disease risk.

## Introduction

Across North America, ticks pose significant threats to the health of humans and animals as vectors of disease agents [1]. Tick surveillance programs have demonstrated expansions of tick species’ geographic distributions, and have increased awareness of the ubiquity of tick-borne pathogens over the last few decades [2]. In some areas, such as in the central Great Plains—defined as Kansas and Oklahoma for the purposes of this paper—tick diversity and abundance vary geographically between east and west or north and south; these contrasts, at least in part, are consequences of tick host preferences and changing tick habitats [3,4]. Peripheral regions within tick species’ ranges, like the central Great Plains, offer opportunities to understand tick range and activity dynamics as expansion occurs across the region.

In the central Great Plains, Oklahoma and Kansas specifically, several established and medically-relevant tick species are present, including Amblyomma americanum*, A. maculatum, Dermacentor albipictus, D. variabilis*, and *Ixodes scapularis* [5]. These species differ in their distribution, host preferences, peak activity periods, habitats, and the pathogens that they transmit [6]; all of these variables must be considered when assessing overall tick risk. For instance, adult *A. americanum* and *I. scapularis* prefer white-tailed deer (*Odocoileus virginianus*) as hosts and forests as habitat, but their peak activity periods are distinct, in spring-summer or fall-winter, respectively [7,8]. Therefore, risk for pathogens transmitted by *I. scapularis* will be higher in fall and winter, whereas those carried by *A. americanum* will be of greater concern in spring and summer.

In this contribution, we develop models to produce detailed “time-and-space” characterizations of environmental conditions that favor questing activity for four medically important tick species that are found across Kansas and Oklahoma (*A. maculatum, D. albipictus, D. variabilis*, and *I. scapularis*). We have previously presented models for *A. americanum*, the most common (i.e., massively dominant) species in tick communities in the region [4,9]. The suite of models presented in this contribution is novel, assessing heterogeneity in distributions of questing ticks in both time and space, using time-specific environmental signatures and explicitly considering sampling effort. Here, we analyze the remaining four tick species that we encountered in our field program (August 2020 to September 2022), including 181 visits to 10 sites across central and eastern Kansas and Oklahoma [4]. This contribution constitutes a first step toward a synthetic view of risk of transmission of tick-borne pathogens to humans, both across space and through time across a broad region.

## Methods

The analyses presented herein were developed via four main methodological steps, as follows. (i) Prepare occurrence and sampling records for each tick species using information obtained from detailed field sampling during 2020–2022; (ii) develop weekly environmental data summaries to include time-specific information on conditions across the study area while associating those conditions with tick occurrence and sampling records; (iii) test for sets of environmental conditions that are related to occurrence and non-occurrence of each tick species; and finally, (iv) develop ecological niche models and use them to create weekly predictions for each tick species. Full elaboration of the workflow employed is provided in a previous analysis of one tick species [9], and in greater detail in a general description of the methods [10]. Scripts to reproduce all analyses are provided at https://github.com/marlonecobos/Tick_KSOK/tree/main/Four_species_ENM.

### Presence-absence data

**Fig 1:**
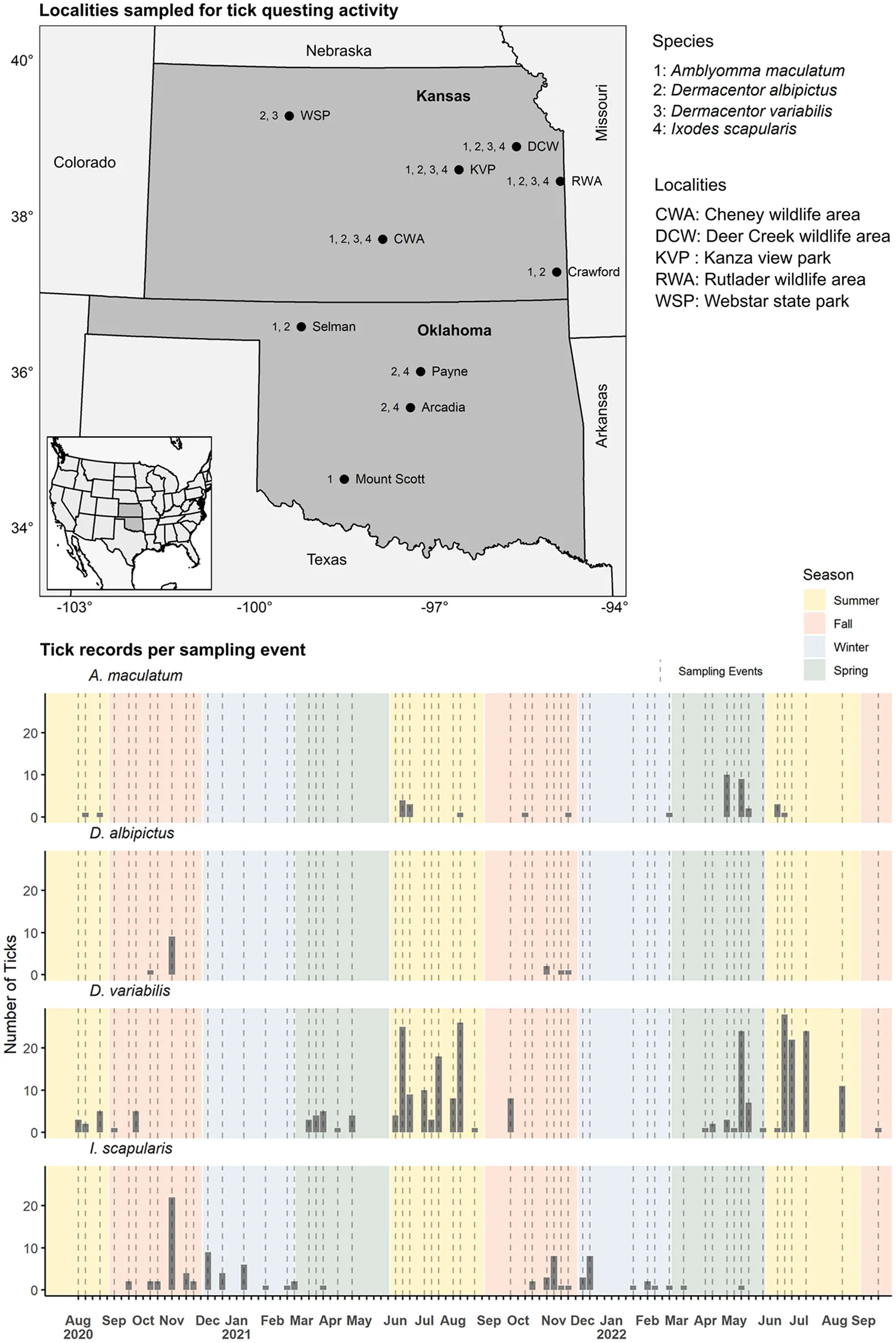
Summary of sampling events (vertical dashed lines) and detections of ticks of four species (*Amblyomma maculatum*, *Dermacentor albipictus*, *D. variabilis*, and *Ixodes scapularis*; gray bars) across the 10 sites sampled as part of our field studies of questing tick behavior. Seasons are shown as green = Spring, yellow = Summer, red = Fall, and blue = Winter. Map data retrieved from GADM (https://gadm.org/).

Occurrence (presence) and non-occurrence (i.e., non-detection in our field surveys) data were obtained from a program of longitudinal sampling at 10 sites across Kansas and Oklahoma, lasting from 1 August 2020–30 September 2022 (Fig 1), and described in detail elsewhere [4]. The sampling included intensive efforts to collect questing ticks in wooded and open areas at each site in the study, using a consistent sampling protocol combining flagging, dragging, and CO_2_ traps. Sampling teams consisted of 3–7 persons, and sampling visits lasted 3–4 hours. Sampling was conducted under conditions considered to be appropriate for tick questing (temperature 2–32ºC, wind speed <15 mi/h, and no precipitation or dew). In all, sampling effort comprised 181 visits across the 10 sites.

From the set of days and sites sampled (Fig 1), we obtained records of presence and apparent absence of questing individuals for each of the four tick species to be treated in this paper (models for *A. americanum* have been presented separately; [9]). For *D. variabilis*, we collected 289 (78.1%) adults and 42 (11.4%) nymphs, so models were developed only for adults of this species. Only adult ticks were collected for *A. maculatum* and *I. scapularis*; only larvae were collected for *D. albipictus*. We assembled data sets in the form of all records of each of the species, to allow analysis of the four species. A presence record is a unique combination of tick species, day, month, year, and site; an absence is also a unique combination of the same fields, but for which the species was not detected in our field surveys. All data processing steps were performed using base functions in R [11].

### Climatic data

We obtained daily climatic data from the Daymet database [12]. We included the following variables in analyses: maximum air temperature (tmax), minimum air temperature (tmin), precipitation (prcp), shortwave radiation (srad), water vapor pressure (wvap), and day length (dayl). Temperature variables were chosen to represent potential limits for questing activity. Precipitation is a variable that represents water availability in an area and, similar to vapor pressure, is a proxy to humidity, which can inform about desiccation risk. Solar radiation and day length represent energy and time available for questing activity. All of these variables have been found to be related in previous studies that explore factors related to tick abundance and activity [13–16].

The climatic layers were cropped to a rectangular extent (33.62–40.00ºN, 103.00–94.5ºW) covering the entire states of Kansas and Oklahoma. All analyses were developed at 1 km spatial resolution, as that is the finest spatial resolution to which the weather data could responsibly be downscaled. Though ticks clearly respond to environmental conditions at finer spatial scales, this coarser-scale analysis provides a view of suitability for questing activity by ticks on a regional extent, rather than at micro-scales, permitting development of geographic-scale views of suitability.

We produced environmental data layers at a temporal resolution of 8 days, averaging over daily values, for the years 2020–2022, as they include temporally the sampling events that produced the tick presence-absence data, and match the temporal grain in our field sampling [4]. The 8-day averaging periods resulted in 46 such sets of data layers per year (i.e., Julian day 1–8, 9–16, ...), although the last period of each year covered only 5–6 days, depending on the year; we used the Julian day for each 8-day average layer for naming layers uniquely. Variable processing including spatial and temporal filtering, and the calculation of averages was done in Google Earth Engine [17].

In a basic exploration of relative independence versus dependence among environmental variables, we calculated Pearson’s correlation coefficients among the 6 original variables, and detected high correlations (i.e., | *r* | > 0.8) between several pairs of variables (S1 File: S1 Table). As a consequence, we used principal components analysis (PCA) to obtain sets of orthogonal predictors from the original variables (S1 File: S2 and S3 Tables). This analysis was applied to the environmental values, characterizing Julian weeks, extracted to the presence-absence data for all species. We then transformed all the Julian-week environmental variables to obtain principal components (PCs) covering January 2020 through December 2022. This step created a dataset that characterized occurrences and non-occurrences in our tick-collection data in environmental dimensions specific in both time and space. All of these raster processing procedures were performed with base R functions and the package terra [18].

### Niche signal exploration

A first analysis step was to test for a niche signal—in effect, a non-random distribution in environmental space as regards which of our sampling events did *versus* did not detect the tick species in question. If the species is present in all samples, or if detections are random with respect to environmental dimensions, then no niche signal is manifested across that time span and study region. Cobos and Peterson [19] presented detailed descriptions of new methods for multivariate (PERMANOVA) and randomization-based univariate tests for such niche signals. The PERMANOVA tests assess whether the position and dispersion of the records of detection are similar to all of the records (detections and non-detections) [19]. Rejecting the PERMANOVA null hypothesis indicates that detections show a different environmental signal compared to all records (the sampling universe).

The univariate tests, on the other hand, assess niche signals via random resampling of the overall dataset to assess the random-distribution null hypothesis directly [19]. Specifically, if one has an overall sample of size *N*, out of which a smaller subset of samples (termed *x*) proved to be positive for a phenomenon (in this case, questing behavior by a particular tick species), then a large number (e.g., in this case 1000) of samples of size *x* are drawn at random from the full set of *N* sampling events to create a null distribution. The mean, median, standard deviation, and range of these replicate samples in terms of environmental data values are then characterized, and the observed value among the real *x* positive samples is compared to that distribution. We performed the multivariate and univariate tests using raw environmental variables as they are directly interpretable compared to PCs. Niche signal explorations using both approaches were developed and interpreted so as to avoid over-reliance on single methodological approaches. Analyses were performed using the R package enmpa [10].

### Model development

Model calibration was done via methods that contrast with the usual approach in ecological niche modeling [20]. This is, instead of presence and background records, we used presence and absence data deriving from our tick sampling program across the two states (detection and non-detection of questing ticks). These data were associated with PC values instead of raw environmental variables to prevent issues from multicollinearity, as PCs are orthogonal axes deriving from the original dimensions. We chose deliberately to include all six of the PCs in our initial analyses, because (1) although the first few PCs generally explain much more of the overall variation, subsequent PCs may respond to important, if more subtle, factors of importance to tick activity; and (2) our analyses include various steps designed to avoid overfitting or overparameterization in our models.

We used logistic regression within a generalized linear model framework (GLMs; family = binomial(link = “logit”) in R). This approach relates positive (detections) and negative (non-detections) occurrence data to environmental predictors to estimate conditional probability of “presence” *versus* “absence.” Evaluations of GLMs based on presence and pseudoabsence data (usually resampled from areas that did not hold presences) using metrics that rely on specificity are not necessarily appropriate because pseudoabsences are assumed to be records of true absence [20]. However, results of tests incorporating specificity can be appropriate and powerful when high-quality presence-absence data are available [9].

Our GLM calibration approach consisted of testing a large suite of candidate models representing all possible combinations of predictors derived from linear and quadratic responses of 6 principal component variables, for a total of 4095 candidate models. All candidate models were created and evaluated for predictive ability using a 10 *k*-fold partitioning approach (i.e., the method to split training and testing data) [20]. Two metrics, area under the curve of the receiver operating characteristic (ROC AUC; [21]) and true skill statistic (TSS; [22]) were used to assess performance and predictive power of candidate models (Note that use of these metrics is appropriate because positive and negative data are available [23]). We also created candidate models with the whole set of data and used the Akaike information criterion (AIC) to assess model goodness-of-fit, penalized by model complexity.

Once all candidate models were fitted and evaluated, we retained the subset of models that passed four filtering steps, following pipelines presented in previous approaches to presence-only niche models [24]. That is, we retained models (1) that had all quadratic predictor coefficients negative (i.e., variable response curves were convex or unimodal, and not concave or bimodal); from among those models, we retained (2) models that had AUC > 0.5, and then kept only (3) those with TSS > 0.4. Finally, (4) we chose as final models only the ones with AIC scores within 2 units of the minimum value among those that had passed the first three filters (i.e., ΔAIC ≤ 2). Model calibration was run in parallel, in a desktop computer (Core Intel Xeon; logical processors: available = 52, used = 32; RAM: available = 128 GB, max used for process = 10 GB), and took less than a minute.

Once we had selected final models, we transferred each of the set of best models separately to all weekly environmental summaries for the sampling period. To create a single consensus prediction per week, we created a weighted average of all best model predictions based on the AIC weights calculated for selected models. This way, models with higher AIC weights (models with better values of fit penalized by complexity) contribute more to the results obtained. All modeling steps were performed using the R package enmpa [10].

### Post-modeling analyses

Given the vagaries of weather and its variation in individual days and weeks, we smoothed weekly predictions using a moving window approach, in which each week was replaced with the average value of the week before, the focal week, and the following week (Cobos et al. 2024). This approach provides a view of results that are less influenced by extreme weather effects during specific weeks. For greater interpretability, we also produced monthly averages from weekly predictions by creating averages of the weekly rasters across the months in which they fall, including partial membership of weeks that overlap between consecutive months.

To identify areas in Kansas and Oklahoma with environmental conditions outside the ranges of conditions represented in the data used to fit models, we used the mobility-oriented parity metric (MOP; [25]). MOP analyses were applied to all environmental summaries for 2020–2022 (i.e., PC fitting data were compared to the PC rasters obtained for every Julian week). Monthly summaries of MOP results were obtained similarly to monthly averages of model predictions; higher values in these summaries indicate that such areas have been outside ranges involved in model fitting for more of the month. MOP analyses were done using the R package mop [26]. All other analyses in this section were done using the R package terra.

## Results

After data preparation and quality-control steps, we had a total of 178 sampling records, which represent a combination of presences and absences of tick questing activity across all of our species (these data are available in full at the code repository). From those sampling records, we derived 16 presences for *A. maculatum*, 9 for *D. albipictus*, 53 for *D. variabilis*, and 31 for *I. scapularis*. The number of absence records for each species is equivalent to 178 minus the number of presence records.

The first principal component explained 67% of the overall variance, and the second and third explained 19% and 10%, respectively (S1 File: S3 Table). As we explored the potential for use of all of the principal components, the full set of components explained all of the variance in the system (S1 File: S3 Table). Principal components 1, 4, and 6 had high loadings by temperature variables (component 4 also had high loadings of day length); components 2 and 3 had high loadings by precipitation and solar radiation; and component 5 was loaded primarily by solar radiation and vapor pressure (S1 File: S2 Table).

### Niche signal tests

The multivariate PERMANOVA showed that, for all species, environmental bias exists when comparing presences *versus* the full data set representing our sampling. This is, we found consistent and statistically significant results in comparisons for *A. maculatum* (F = 2.58, P < 0.05), *D. albipictus* (F = 3.25, P < 0.05), *D. variabilis* (F = 4.79, P < 0.05), and *I. scapularis* (F = 4.34, P < 0.05). The univariate tests for non-random distribution of detections of each species with respect to sampled environmental conditions indicated significant differences from null expectations in most (but not all) of the environmental dimensions that we assessed (S1 File: S1 Fig, S4–S7 Tables). Some were as expected, given the phenological variation among species that we have documented elsewhere [4]: for example, the two fall-winter tick species (i.e., *I. scapularis*, *D. albipictus*) are found questing under conditions of shorter day length, whereas the two spring-summer tick species (i.e., *D. variabilis*, *A. maculatum*) are found questing under longer day length, as compared with null expectations from the sampling scheme.

In fact, these same differences hold for precipitation and vapor pressure, with fall-winter ticks occurring under drier conditions and spring-summer ticks occurring under wetter conditions compared to null expectations. The relationships of the two temperature variables and solar radiation showed similar contrasts between fall-winter and spring-summer tick species, but the rarer species (*D. albipictus*, *A. maculatum*) were more equivocal in the significance of their niche signal, likely owing to lower statistical power.

### Models of questing activity

**Fig 2:**
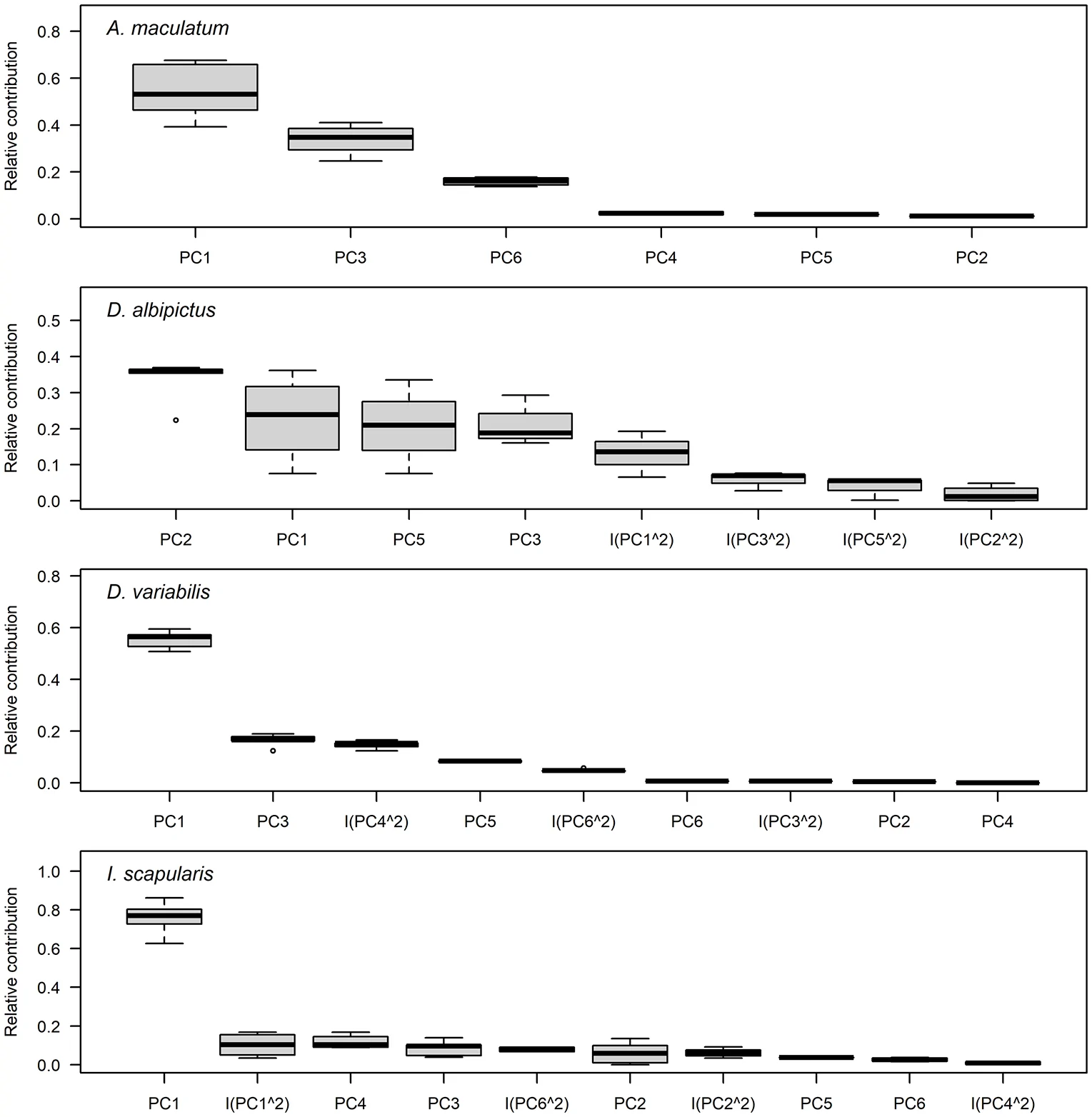
Summary of predictor contribution in models picked during model selection for the four species of ticks in this study. Box plots are used to represent contributions as multiple models met the selection criteria per species.

The numbers of models that passed the selection criteria varied among species, six for *A. maculatum*, five for *D. albipictus*, five for *D. variabilis*, and 14 for *I. scapularis* (S1 File: S8–S11 Tables). Models selected used linear and quadratic forms of the variables, with higher-order components (i.e., 5 and 6) contributing more to models than some of the components that explained greater amounts of variance (Fig 2). Variable response curves of selected models showed similar patterns among alternate models for the same species (S1 File: S2–S5 Figs). Highest variability in response curves was found in models selected for *D. albipictus* (component 2; S1 File: S3 Fig) and for *Ixodes scapularis* (components 1, 2, 4, and 6; S1 File: S5 Fig).

**Fig 3:**
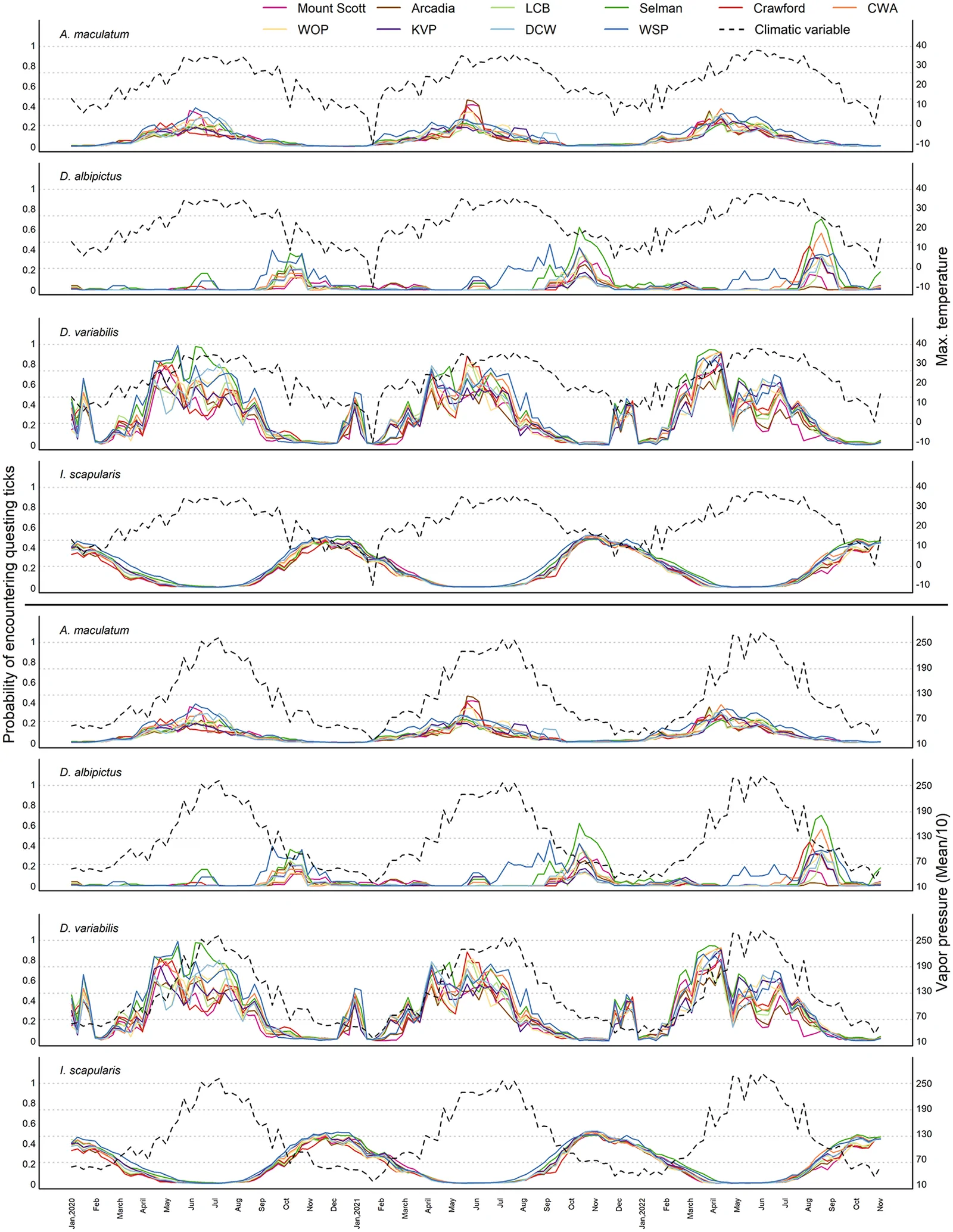
Temporal traces across all of 2020–2022 for probability of questing for four species of ticks at 10 sites in Kansas and Oklahoma. Probability of questing at each site is shown as a solid line with a different color. For the purposes of illustration and interpretation, we provide a visualization of seasonal variation in average maximum temperature and vapor pressure across all sites, shown as dotted lines. Temperature and vapor pressure (as a proxy of humidity) were chosen for this visualization in view of their known general importance in determining activity patterns of ticks (Requena-García et al. 2017).

**Fig 4:**
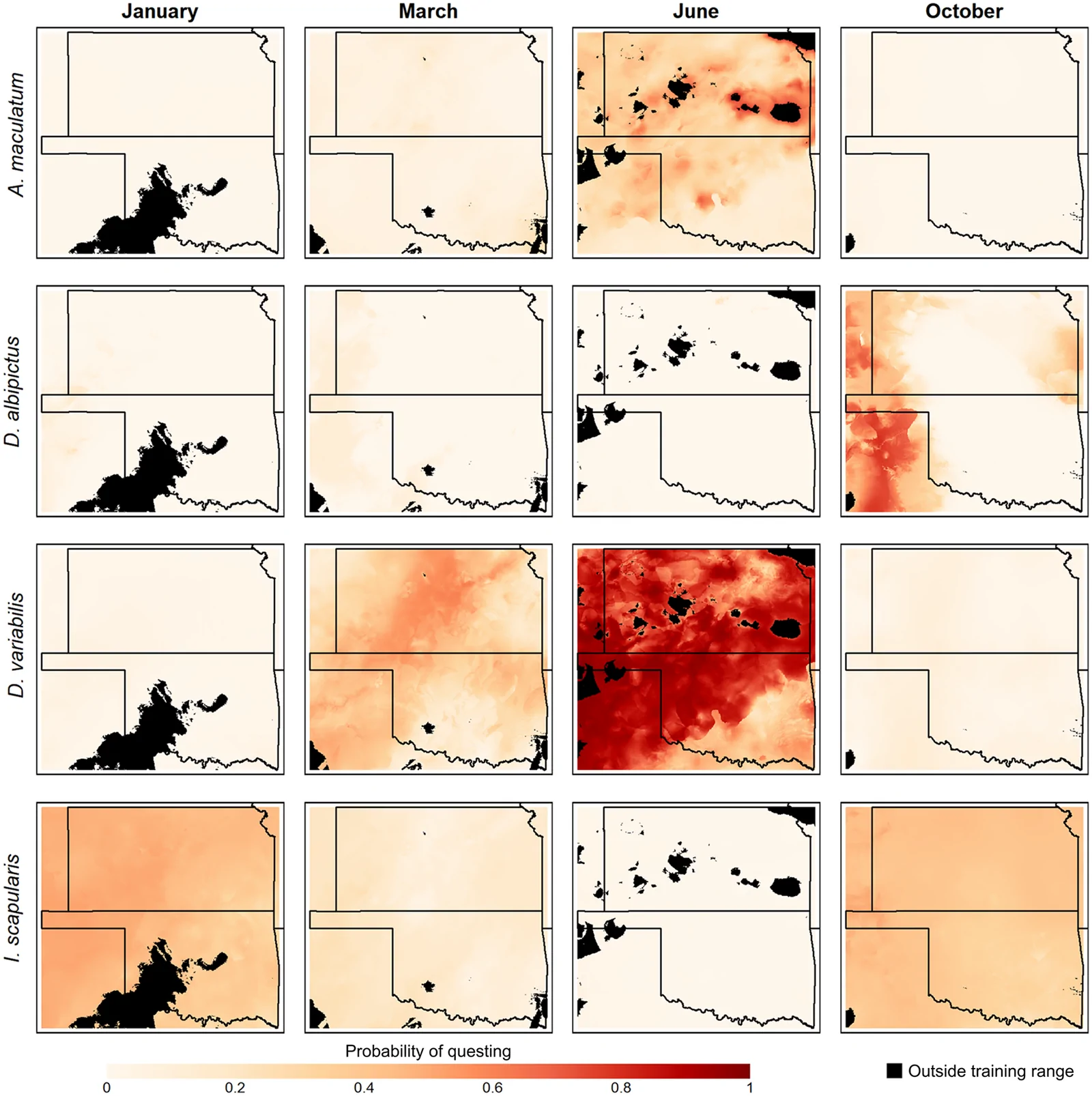
Summary of time-specific ecological niche model predictions for four example weeks in 2021, for four tick species. Areas shown in black were identified as presenting conditions outside model training ranges in that time period via MOP analyses, and shown where model predictions could be uncertain due to model extrapolation. Map data retrieved from GADM (https://gadm.org/).

The four species under analysis in this contribution show at least two distinct signatures of seasonality of questing behavior, as can be appreciated in the form of Spring-Summer peaks of predicted probability of questing for *A. maculatum* and *D. variabilis*, contrasting with Fall peaks in *D. albipictus* and Fall-Winter peaks in *I. scapularis* (Fig 3; analyzed in detail elsewhere; [4]). These seasonal patterns of variation in probability of questing, as reflected in model predicted signatures through time, echo the phenology of the species (Figs 3,4; [4]). Of particular note is the covariation between predicted questing patterns and key climatic variables, as is illustrated in Fig 3 as well.

The ecological niche models developed for the four tick species reflected these same contrasts between phenological types of tick species (i.e., fall-winter *versus* spring-summer; Fig 4; S2–S9 Files). That is, *D. variabilis* begins to rise in activity in Spring, and reaches a maximum in the Summer; *A. maculatum* is more strictly focused in Summer. Similarly, larvae of *D. albipictus* are active in the Fall, whereas *I. scapularis* is active in Fall and Winter. Extrapolative conditions (i.e., outside training ranges) were found in southwestern Oklahoma in Winter in terms of PC6, and were in scattered parts of Kansas and western Oklahoma in Summer in terms of different combinations of PC1, PC2, PC3, PC5, and PC6 (Fig 4; S1 File: S6 Fig.). Areas presenting extrapolative conditions were generally small, and did not cover much of the study area during periods of higher probability of questing for three of the four species (S2–S4 Files, S6–S8 Files). Slightly larger areas with extrapolative conditions were found for *I. scapularis* during winter conditions (S5 and S9 Files).

## Discussion

### New modeling approaches

This study explores the potential strengths of a suite of new ecological niche modeling approaches, as applied to four tick species in the central Great Plains region. We incorporate three major changes from the typical analytical approaches in distributional ecology: (1) time-specific environmental values assigned to individual sampling events, (2) use of information directly from the sampling that produced the positive and negative records of the species, and (3) constraint of the response shapes in the models to be convex and more consistent with current understanding of what fundamental ecological niches should be like. More detail is provided below on each of these points.

Typically, ecological niche modeling applications use a single environmental value for each site, neglecting the sometimes-extreme environmental variation that is manifested at a site in the course of days, years, or decades [27]. Early explorations of the idea of time-specific niche modeling approaches showed the greater specificity that can be achieved by avoiding the fallacy that an average can represent the variation that goes into the average value adequately [28]. A later paper explored contrasts between time-specific and time-averaged approaches in distributional ecology, and provided methodological protocols (although they were computationally intensive) for handling presence-only data in such analyses [27].

The analyses presented herein improve on those previous time-specific methods by direct and full incorporation of information from the sampling that produced the set of positive records of each species [9]. That is, in the analyses presented herein, our models represent statistical contrasts between sampling events that did and did not detect a given species. In this sense, the negative data in our analyses are not “pseudo-absence” data that are assumed to represent absence of the species [20]; rather, they are sampling events with field methods equivalent to those that yielded presence records, but on which the species in question was not encountered. In this sense, we have refined the negative data considerably from the usual approach in distributional ecological analyses, which is likely to illuminate the niche signal recovered in the modeling process considerably.

Finally, we pursue yet another improvement to the approach of testing models calibrated under different parameter settings that began with the work of Warren and Seifert [29], and refined and extended considerably with the development of the KUENM R package [24]. That is, for a number of years, model selection has included consideration of model significance and predictive performance, as well as model simplicity. Here, following other recent analyses (e.g., [30]), we add one more consideration: that model response types “look like” fundamental ecological niches, in that they are unimodal with respect to each environmental dimension, as well as convex in multivariate space [31–33].

### Diversity of tick questing activity patterns

As witnessed in the present paper, tick questing behavior varies across time, space, and by species. Ticks are known to prefer particular climatic variables (e.g., relative humidity, ambient temperature), but can survive weather extremes [34]. The latter scenario is critical for long-term tick survival; however, their preferences dictate their peak activity or questing periods, which relate or correspond to times at which interaction of ticks with hosts may be highest, which in turn drives spatiotemporal patterns of tick-borne disease risk. Across the four species presented here, variation in questing patterns was dramatic, which was unsurprising based on prior research in other regions of North America and the seasonal variation expected for the central Great Plains [35–37].

For warm-season ticks, such as *A. maculatum* and *D. variabilis*, their questing periods peaked in the summer months, in general (see GIF animations S2 and S4 Files, respectively). These two species are known to enter diapause in cooler months of the year, overwintering as immature stages; they may have an extended life cycle duration in comparison to other tick species. Once temperatures rise above ~20°C, immature stages of these species are more likely to be questing for hosts; temperature is known to be an important predictor of their survival [38].

In contrast, cooler-season ticks (larval *D. albipictus* and *I. scapularis*) have peak activity periods in the late fall or winter (see GIF animations S3 and S5 Files, respectively, for greater detail). That is, the activity of these two species in the central Great Plains is closely tied to declining values of temperature and vapor pressure (humidity) in the Fall, and neither was ever detected under conditions of higher temperature or humidity. We note that *D. albipictus* was only detected in larval stages, as it appears that all other life stages remain on the host [39]. These ticks are known to pause activity under more extreme conditions [40].

In the central Great Plains, these temperature-dependent behaviors are expected to vary based on specific location. Prior work has demonstrated that environmental conditions in some parts of this region support ticks year-round [41], which is not the case across the entirety of the central Great Plains [8,42]. As certain vegetation types (e.g., forest) and reservoir hosts (e.g., deer) are increasingly able to survive in historically non-endemic tick regions of the central Great Plains, novel tick populations may enter and establish [43,44], or perhaps they have been there, but at low population levels, and are now able to increase. Most of the study sites in the present study were in the eastern or central regions of the two states; however, if the westernmost parts of Oklahoma and Kansas are considered, temperature, relative humidity, and terrain are quite different. More work is warranted to compare and identify additional factors that can lead to tick expansion in this region so more accurate risk modeling can occur for areas with emerging populations of medically-relevant ticks.

## Conclusion and public health implications

This paper presents a complex and varied view of tick questing activity through the year and across the central Great Plains, across four tick species. The fifth tick species that is widespread in the region, *A. americanum* is treated in a separate contribution [9]. This complexity of questing activity by a diversity of tick species has important implications for public health in the region. A large subsample of the same ticks that were collected as part of the work reported herein have been tested for a battery of bacterial pathogens (e.g., *Borrelia*, *Anaplasma*, *Ehrlichia*); results of those tests with respect to pathogens will be reported elsewhere (K. Duncan et al. in prep.). Overall, the picture is one of extreme complexity of multiple species of pathogens transmitted by multiple species of ticks, which in turn have diverse life histories, ecological niches, phenologies, seasonalities, and spatiotemporal distributional patterns.

## Supporting information

S1 File Supplementary tables and figures. Tables and figures are presented in the order in which they appear in the text; all tables are listed first, then figures.
(DOCX)

S2 File Animated weekly predictions of *Amblyomma maculatum* questing activity, highlighting areas with conditions outside the training range of models. (GIF)

S3 File Animated weekly predictions of *Dermacentor albipictus* questing activity, highlighting areas with conditions outside the training range of models. (GIF)

S4 File Animated weekly predictions of *Dermacentor variabilis* questing activity, highlighting areas with conditions outside the training range of models. (GIF)

S5 File Animated weekly predictions of *Ixodes scapularis* questing activity, highlighting areas with conditions outside the training range of models. (GIF)

S6 File Animated monthly average predictions of *Amblyomma maculatum* questing activity, highlighting areas with conditions outside the training range of models. The mean of weekly results from mobility-oriented parity analysis is used to depict non-analogous conditions per month.
(GIF)

S7 File Animated monthly average predictions of *Dermacentor albipictus* questing activity, highlighting areas with conditions outside the training range of models. The mean of weekly results from mobility-oriented parity analysis is used to depict non-analogous conditions per month.
(GIF)

S8 File Animated monthly average predictions of *Dermacentor variabilis* questing activity, highlighting areas with conditions outside the training range of models. The mean of weekly results from mobility-oriented parity analysis is used to depict non-analogous conditions per month.
(GIF)

S9 File Animated monthly average predictions of *Ixodes scapularis* questing activity, highlighting areas with conditions outside the training range of models. The mean of weekly results from mobility-oriented parity analysis is used to depict non-analogous conditions per month.
(GIF)
