# Supplementary material for: Spatiotemporal dynamics of questing activity by four tick species in the central Great Plains
Source: PLoS One. 2026 Sep 1;21(9):e0351160. doi: 10.1371/journal.pone.0351160 (PMC13533356; doi:10.1371/journal.pone.0351160)
Supplement: Supplementary file 1 [file pone.0351160.s001.docx]

**Supplementary Information**

**S1 Table. Pearson correlations among environmental variables.** Correlations greater than 0.5 or less than -0.5 are indicated in bold.

|  | Mean day length | Mean precipitation | Mean solar radiation | Mean maximum temperature | Mean minimum temperature | Mean vapor pressure |
| --- | --- | --- | --- | --- | --- | --- |
| Mean day length | 1 | – | – | – | – | – |
| Mean precipitation | 0.28 | 1 | – | – | – | – |
| Mean solar radiation | **0.75** | -0.13 | 1 | – | – | – |
| Mean maximum temperature | **0.73** | -0.001 | **0.73** | 1 | – | – |
| Mean minimum temperature | **0.82** | 0.15 | **0.64** | **0.95** | 1 | – |
| Mean vapor pressure | **0.80** | 0.12 | **0.58** | **0.91** | **0.97** | 1 |

**S2 Table. Raw climatic variable loadings on each of the 6 principal components.** Bolded values represent the most relevant variables for each principal component (PC).

|  | PC1 | PC2 | PC3 | PC4 | PC5 | PC6 |
| --- | --- | --- | --- | --- | --- | --- |
| Mean day length | 0.456 | -0.026 | 0.379 | **0.634** | 0.448 | -0.211 |
| Mean precipitation | 0.121 | **0.836** | **0.461** | -0.235 | -0.139 | -0.014 |
| Mean solar radiation | 0.359 | **-0.492** | **0.553** | -0.194 | **-0.502** | 0.183 |
| Mean maximum temperature | **0.474** | -0.099 | -0.189 | **-0.597** | 0.255 | **-0.556** |
| Mean minimum temperature | **0.482** | 0.098 | -0.262 | -0.138 | 0.293 | **0.765** |
| Mean vapor pressure | 0.438 | 0.199 | -0.483 | 0.361 | **-0.614** | -0.167 |

**S3 Table. Summary of the variance explained by each of the principal components (PC).**

|  | PC1 | PC2 | PC3 | PC4 | PC5 | PC6 |
| --- | --- | --- | --- | --- | --- | --- |
| Standard deviation | 2.004 | 1.076 | 0.762 | 0.402 | 0.264 | 0.120 |
| Proportion of Variance | 0.670 | 0.193 | 0.097 | 0.027 | 0.012 | 0.002 |
| Cumulative Proportion | 0.670 | 0.862 | 0.959 | 0.986 | 0.997 | 1 |

**S4 Table. Summaries of the univariate tests for the existence of a distinguishable environmental bias (i.e., ecological niche) of *Amblyomma maculatum*.** “Higher” and “lower” indicate that the observed value of the tick occurrences with regard to the relevant environmental variable is located in the upper or lower 2.5% of the null distribution, respectively. H0: null hypothesis; SD: standard deviation.

| Variable | H0 mean | H0 median | H0 SD | H0 range | Positive mean vs null | Positive median vs null | Positive SD vs null | Positive range vs null |
| --- | --- | --- | --- | --- | --- | --- | --- | --- |
| Mean day length | rejected | rejected | accepted | accepted | higher | higher | indistinct | indistinct |
| Mean precipitation | rejected | rejected | accepted | rejected | higher | higher | indistinct | higher |
| Mean solar radiation | rejected | accepted | accepted | accepted | higher | indistinct | indistinct | indistinct |
| Mean maximum temperature | rejected | rejected | accepted | accepted | higher | higher | indistinct | indistinct |
| Mean minimum temperature | rejected | rejected | accepted | accepted | higher | higher | indistinct | indistinct |
| Mean vapor pressure | rejected | rejected | accepted | accepted | higher | higher | indistinct | indistinct |

**S5 Table. Summaries of the univariate tests for the existence of a distinguishable environmental bias (i.e., ecological niche) of *Dermacentor albipictus*.** “Higher” and “lower” indicate that the observed value of the tick occurrences with regard to the relevant environmental variable is located in the upper or lower 2.5% of the null distribution, respectively. H0: null hypothesis; SD: standard deviation.

| Variable | H0 mean | H0 median | H0 SD | H0 range | Positive mean vs null | Positive median vs null | Positive SD vs null | Positive range vs null |
| --- | --- | --- | --- | --- | --- | --- | --- | --- |
| Mean day length | rejected | rejected | rejected | rejected | lower | lower | lower | lower |
| Mean precipitation | rejected | accepted | rejected | rejected | lower | indistinct | lower | lower |
| Mean solar radiation | rejected | accepted | rejected | rejected | lower | indistinct | lower | lower |
| Mean maximum temperature | accepted | accepted | rejected | rejected | indistinct | indistinct | lower | lower |
| Mean minimum temperature | rejected | accepted | rejected | rejected | lower | indistinct | lower | lower |
| Mean vapor pressure | rejected | accepted | rejected | rejected | lower | indistinct | lower | lower |

**S6 Table. Summaries of the univariate tests for the existence of a distinguishable environmental bias (i.e., ecological niche) of *Dermacentor variabilis*.** “Higher” and “lower” indicate that the observed value of the tick occurrences with regard to the relevant environmental variable is located in the upper or lower 2.5% of the null distribution, respectively. H0: null hypothesis; SD: standard deviation.

| Variable | H0 mean | H0 median | H0 SD | H0 range | Positive mean vs null | Positive median vs null | Positive SD vs null | Positive range vs null |
| --- | --- | --- | --- | --- | --- | --- | --- | --- |
| Mean day length | rejected | rejected | rejected | rejected | higher | higher | lower | lower |
| Mean precipitation | rejected | rejected | accepted | accepted | higher | higher | indistinct | indistinct |
| Mean solar radiation | rejected | rejected | rejected | accepted | higher | higher | lower | indistinct |
| Mean maximum temperature | rejected | rejected | rejected | rejected | higher | higher | lower | lower |
| Mean minimum temperature | rejected | rejected | rejected | rejected | higher | higher | lower | lower |
| Mean vapor pressure | rejected | rejected | rejected | rejected | higher | higher | lower | lower |

**S7 Table. Summaries of the univariate tests for the existence of a distinguishable environmental bias (i.e., ecological niche) of *Ixodes scapularis*.** “Higher” and “lower” indicate that the observed value of the tick occurrences with regard to the relevant environmental variable is located in the upper or lower 2.5% of the null distribution, respectively. H0: null hypothesis; SD: standard deviation.

| Variable | H0 mean | H0 median | H0 SD | H0 range | Positive mean vs null | Positive median vs null | Positive SD vs null | Positive range vs null |
| --- | --- | --- | --- | --- | --- | --- | --- | --- |
| Mean day length | rejected | rejected | rejected | rejected | lower | lower | lower | lower |
| Mean precipitation | rejected | accepted | rejected | accepted | lower | indistinct | lower | indistinct |
| Mean solar radiation | rejected | rejected | rejected | accepted | lower | lower | lower | indistinct |
| Mean maximum temperature | rejected | rejected | rejected | rejected | lower | lower | lower | lower |
| Mean minimum temperature | rejected | rejected | rejected | accepted | lower | lower | lower | indistinct |
| Mean vapor pressure | rejected | rejected | rejected | rejected | lower | lower | lower | lower |

**S8 Table. “Best” models selected in the process of model calibration and evaluation for *Amblyomma maculatum*.** “^2” indicates quadratic terms in a model. Values for AUC, sensitivity, specificity, and TSS are averages deriving from the 10 k-fold evaluation process. Threshold indicates the value used to test sensitivity and specificity of the model. AUC = area under the receiver operative characteristic curve; TSS = true skill statistic; AIC = Akaike information criterion; ΔAIC = delta AIC.

| Predictors | Threshold | ROC AUC | Sensitivity | Specificity | TSS | AIC | Delta AIC | AIC weight |
| --- | --- | --- | --- | --- | --- | --- | --- | --- |
| PC1, PC3, PC6 | 0.136 | 0.762 | 0.900 | 0.772 | 0.672 | 98.070 | 1.961 | 0.111 |
| PC1, PC3 | 0.110 | 0.793 | 0.950 | 0.753 | 0.703 | 96.109 | 0.000 | 0.296 |
| PC1, PC3, PC4 | 0.158 | 0.776 | 0.850 | 0.846 | 0.696 | 96.672 | 0.563 | 0.223 |
| PC1, PC2, PC3, PC6 | 0.131 | 0.790 | 0.900 | 0.810 | 0.710 | 97.821 | 1.712 | 0.126 |
| PC1, PC3, PC4, PC6 | 0.125 | 0.795 | 0.950 | 0.740 | 0.690 | 97.948 | 1.839 | 0.118 |
| PC1, PC3, PC5, PC6 | 0.104 | 0.780 | 0.950 | 0.741 | 0.691 | 97.818 | 1.709 | 0.126 |

**S9 Table. “Best” models selected in the process of model calibration and evaluation for *Dermacentor albipictus*.** “^2” indicates quadratic terms in a model. Values for AUC, sensitivity, specificity, and TSS are averages deriving from the 10 k-fold evaluation process. Threshold indicates the value used to test sensitivity and specificity of the model. AUC = area under the receiver operative characteristic curve; TSS = true skill statistic; AIC = Akaike information criterion; ΔAIC = delta AIC.

| Predictors | Threshold | ROC AUC | Sensitivity | Specificity | TSS | AIC | Delta AIC | AIC weight |
| --- | --- | --- | --- | --- | --- | --- | --- | --- |
| PC1, PC2, PC3, PC5 | 0.136 | 0.877 | 1.000 | 0.836 | 0.836 | 48.201 | 1.648 | 0.133 |
| PC1, PC2, PC3, PC1^2, PC2^2, PC3^2 | 0.138 | 0.867 | 1.000 | 0.817 | 0.817 | 48.262 | 1.709 | 0.129 |
| PC1, PC2, PC5, PC1^2, PC2^2, PC5^2 | 0.148 | 0.865 | 1.000 | 0.818 | 0.818 | 48.021 | 1.467 | 0.146 |
| PC2, PC3, PC5, PC2^2, PC3^2, PC5^2 | 0.140 | 0.845 | 1.000 | 0.754 | 0.754 | 46.554 | 0.000 | 0.304 |
| PC1, PC2, PC3, PC5, PC1^2, PC2^2, PC3^2, PC5^2 | 0.108 | 0.877 | 1.000 | 0.830 | 0.830 | 46.662 | 0.109 | 0.288 |

**S10 Table. “Best” models selected in the process of model calibration and evaluation for *Dermacentor variabilis*.** “^2” indicates quadratic terms in a model. Values for AUC, sensitivity, specificity, and TSS are averages deriving from the 10 k-fold evaluation process. Threshold indicates the value used to test sensitivity and specificity of the model. AUC = area under the receiver operative characteristic curve; TSS = true skill statistic; AIC = Akaike information criterion; ΔAIC = delta AIC.

| Predictors | Threshold | ROC AUC | Sensitivity | Specificity | TSS | AIC | Δ AIC | AIC weight |
| --- | --- | --- | --- | --- | --- | --- | --- | --- |
| PC1, PC3, PC5, PC4^2, PC6^2 | 0.258 | 0.847 | 0.8833 | 0.776 | 0.659 | 152.78 | 0.000 | 0.355 |
| PC1, PC2, PC3, PC5, PC4^2, PC6^2 | 0.227 | 0.847 | 0.9033 | 0.745 | 0.648 | 154.39 | 1.610 | 0.159 |
| PC1, PC3, PC4, PC5, PC4^2, PC6^2 | 0.183 | 0.828 | 0.9433 | 0.690 | 0.633 | 154.78 | 1.998 | 0.131 |
| PC1, PC3, PC5, PC6, PC4^2, PC6^2 | 0.218 | 0.843 | 0.920 | 0.721 | 0.641 | 154.16 | 1.381 | 0.178 |
| PC1, PC3, PC5, PC3^2, PC4^2, PC6^2 | 0.361 | 0.844 | 0.817 | 0.853 | 0.670 | 154.18 | 1.395 | 0.177 |

**S11 Table. “Best” models selected in the process of model calibration and evaluation for *Ixodes scapularis*.** “^2” indicates quadratic terms in a model. Values for AUC, sensitivity, specificity, and TSS are averages deriving from the 10 k-fold evaluation process. Threshold indicates the value used to test sensitivity and specificity of the model. AUC = area under the receiver operative characteristic curve; TSS = true skill statistic; AIC = Akaike information criterion; ΔAIC = delta AIC.

| Predictors | Threshold | ROC AUC | Sensitivity | Specificity | TSS | AIC | Δ AIC | AIC weight |
| --- | --- | --- | --- | --- | --- | --- | --- | --- |
| PC1, PC3 | 0.214 | 0.839 | 0.942 | 0.796 | 0.737 | 132.812 | 1.856 | 0.048 |
| PC1, PC2, PC3 | 0.196 | 0.844 | 0.967 | 0.782 | 0.749 | 131.161 | 0.205 | 0.109 |
| PC1, PC3, PC4 | 0.203 | 0.840 | 0.942 | 0.782 | 0.724 | 131.576 | 0.620 | 0.089 |
| PC1, PC2, PC4 | 0.211 | 0.837 | 0.942 | 0.823 | 0.765 | 132.672 | 1.716 | 0.051 |
| PC1, PC3, PC4, PC6 | 0.180 | 0.831 | 0.975 | 0.773 | 0.748 | 131.437 | 0.481 | 0.095 |
| PC1, PC2, PC4, PC6 | 0.214 | 0.833 | 0.942 | 0.789 | 0.731 | 131.889 | 0.933 | 0.076 |
| PC1, PC2, PC4, PC5 | 0.207 | 0.834 | 0.942 | 0.769 | 0.711 | 132.915 | 1.959 | 0.046 |
| PC1, PC2, PC3, PC4 | 0.217 | 0.843 | 0.942 | 0.804 | 0.746 | 132.516 | 1.560 | 0.056 |
| PC1, PC2, PC4, PC5, PC6 | 0.228 | 0.843 | 0.967 | 0.783 | 0.750 | 131.971 | 1.015 | 0.073 |
| PC1, PC2, PC3, PC4, PC6 | 0.234 | 0.833 | 0.942 | 0.797 | 0.738 | 132.614 | 1.658 | 0.053 |
| PC1, PC2, PC1^2, PC2^2 | 0.276 | 0.820 | 0.908 | 0.815 | 0.724 | 130.956 | 0.000 | 0.121 |
| PC1, PC2, PC4, PC1^2, PC2^2, PC4^2 | 0.284 | 0.842 | 0.942 | 0.831 | 0.772 | 132.052 | 1.096 | 0.070 |
| PC1, PC2, PC6, PC1^2, PC2^2, PC6^2 | 0.269 | 0.826 | 0.908 | 0.783 | 0.692 | 132.164 | 1.208 | 0.066 |
| PC1, PC2, PC4, PC6, PC1^2, PC2^2, PC4^2, PC6^2 | 0.275 | 0.837 | 0.908 | 0.803 | 0.711 | 132.893 | 1.937 | 0.046 |

**S1 Fig. Summary of univariate tests of niche signal (i.e., non-random distribution with respect to availability of sampled conditions) for four tick species with respect to six climatic parameters.** Gray bars indicate frequency of values among 1000 null resamplings from the available data; black vertical dashed bars indicate the 2.5th and 97.5th percentiles of the null distribution. Vertical red line indicates the observed value.

**S2 Fig. Variable response curves and summary of all forms of predictors relative contribution used in models of *Amblyomma maculatum*.**

**S3 Fig. Variable response curves and summary of all forms of predictors relative contribution used in models of *Dermacentor albipictus*.**

**S4 Fig. Variable response curves and summary of all forms of predictors relative contribution used in models of *Dermacentor variabilis*.**

**S5 Fig. Variable response curves and summary of all forms of predictors relative contribution used in models of *Ixodes scapularis*.**

**S6 Fig. Areas in Kansas and Oklahoma with environmental conditions outside training ranges, towards low and high ends of principal components for example weeks in 2021.** Results correspond to examples shown in Fig. 4, and were obtained using the mobility-oriented parity (MOP) metric by defining “detailed” for the parameter “type”.
