# Supplementary material for: Spatiotemporal dynamics of questing activity by four tick species in the central Great Plains
Source: PLoS One. 2026 Sep 1;21(9):e0351160. doi: 10.1371/journal.pone.0351160 (PMC13533356; doi:10.1371/journal.pone.0351160)
Supplement: Supplementary file 2 [file pone.0351160.s011.docx]

**Response to Editor and Reviewers:**

**Spatiotemporal dynamics of questing activity by four tick species in the central Great Plains**

Dear Dr. Brissette,

Thank you for your editorial consideration of the manuscript, “Spatiotemporal dynamics of questing activity by four tick species in the central Great Plains” (PONE-D-25-22057). We appreciate the useful comments and suggestions provided by the reviewers. We have made appropriate clarifications and modifications to our original manuscript in response to those comments and suggestions. We believe that our manuscript has improved substantially, and we hope that you will now find it suitable for publication in PLOS ONE.

Please, find below detailed responses to the comments of the reviewers. Line numbers refer to those of the document without tracked changes.

Sincerely,

The authors

------------------------------------------------------

**Editorial comments:**

Comment: Please ensure that your manuscript meets PLOS ONE's style requirements, including those for file naming.

*Response: Done.*

Comment: “This material is based upon work supported by the National Science Foundation under Grant No. OIA-1920946.” Please state: "The funders had no role in study design, data collection and analysis, decision to publish, or preparation of the manuscript." Please include this amended Role of Funder statement in your cover letter; we will change the online submission form on your behalf.

*Response: Done.*

Comment: We note that Figures 4 and S5 - S8 in your submission contain map images which may be copyrighted. All PLOS content is published under the Creative Commons Attribution License (CC BY 4.0), which means that the manuscript, images, and Supporting Information files will be freely available online, and any third party is permitted to access, download, copy, distribute, and use these materials in any way, even commercially, with proper attribution. For these reasons, we cannot publish previously copyrighted maps or satellite images created using proprietary data, such as Google software (Google Maps, Street View, and Earth). For more information, see our copyright guidelines: http://journals.plos.org/plosone/s/licenses-and-copyright.

*Response: All the spatial elements representing administrative areas were downloaded from the GADM database (*[*https://gadm.org/*](https://gadm.org/)*). Please see the text in the license below (*[*https://gadm.org/license.html*](https://gadm.org/license.html)*):*

*The data are freely available for academic use and other non-commercial use. Redistribution or commercial use is not allowed without prior permission.*

*Using the data to create maps for publishing of academic research articles is allowed. Thus you can use the maps you made with GADM data for figures in articles published by PLoS, Springer Nature, Elsevier, MDPI, etc. You are allowed (but not required) to publish these articles (and the maps they contain) under an open license such as CC-BY as is the case with PLoS journals and may be the case with other open access articles. Data for the following countries is covered by a different license Austria: Creative Commons Attribution-ShareAlike 2.0 (source: Government of Austria).*

------------------------------------------------------

**Reviewer #1:**

General comment: this manuscript should be restructured to be explicitly either i,) a methods paper with a practical example, or ii.) practical medical entomology utilizing a novel method. It's currently a hybrid of the two, greatly complicating the scientific "story."

*Response: Thank you for your comments and suggestions. We have made changes in our text to structure this work as a study using novel methods.*

Comment: Pg 6. Justifications/references for the choice of these environmental parameters (esp. srad and wvap) should be included.

*Response: We have included justification for the inclusion of these variables in our study. Line 106.*

Comment: Pg 6. Brief mention should be made of (presumed) cluster usage, and general run times for these models to support potential replications.

*Response: We have included details on the computing capacity used to perform analysis. Lines 194–196.*

Comment: Pg. 9 "We created a weighed average of all best...." This appears to be an ensemble model; the aggregation choices should be detailed and justified.

*Response: This is not exactly an ensemble model as in packages like biomod2, but similar and only using one algorithm. We have extended our description of the process to make it clear. Lines 198-202.*

Comment: Pg. 10. "Post-modeling analyses" The choice of smoother windows here very likely has a major impact on model outputs. Sensitivity analyses should be reported.

*Response: Since the smoother window was applied to predictions, model performance is not necessarily affected. This approach does affect the way predictions look, and is intended to provide a view of results that are not overly influenced by extreme weather events in certain weeks. We have added these ideas to our text to make it clear for the readers. Lines 208–209.*

Comment: Pg 12. First paragraph. This is the first mention of specific PCs. The complex interpretability of these components needs to be made explicit. Secondly, the utility of higher-order PCs beyond 3-4 is very unusual and should be commented on. Lastly, the nature of the extrapolation regions needs further detail (which parameters were outside the raster datasets?).

*Response: We have included mentions of the PCs in previous parts of the document when we refer to predictors to clarify when they were used and how, and when they were not used. Lines 126–128, 152–154, 162–168.*

*We understand that using high order PCs is not common in ecological niche modeling applications. The reason is that PCAs are usually applied as a tool to reduce dimensionality. In our case, a PCA was used not to reduce dimensions, but to characterize all the variance in new orthogonal (non-collinear) dimensions. We have included a brief mention of this in our manuscript (Lines 162-168). As such, all PCs were used in our exploration because all of them represent unique aspects of environmental variation that can be of relevance to characterize the phenomenon of interest. This is demonstrated by the results from our data-informed model selection process, which shows that selected models tend to use most of the PCs, even the ones of high-order (new Fig. 2).*

*We have added further details about extrapolation risk analysis and results in the document. Lines 292–299.*

Comment: Pg 14-15. The dynamics displayed in Fig 4 are a core part of this manuscript, yet the discussion as written doesn't really engage with the underlying drivers of presence/true-absence. Wherever possible this section should be reconsidered to incorporate references to bio-ecology beyond "warm-season" vs "cold-season" ticks.

*Response: We have expanded our discussion to address this comment. Lines 359–365.*

Comment: Pg. 9 "Once all candidate models...." A diagram showing the model-selection processes would be an important addition.

*Response: Thanks for the suggestion. The model selection process followed here was described in detail in the paper that presented the methods and R package we used, as well as in a previous study for the Lone Star tick. We prefer not to include such a figure, to avoid repetition and leave more space for figures relevant to the topic of this paper. Instead we added more clear references to previous publications using this methodology. Lines 65-68.*

Comment: Pg 10. Figure 2. This figure has limited utility and could be moved to supplemental utility.

*Response: Agree. We have moved this figure to Supplementary information. Instead, we included a summary of predictor contribution to models for all species. To help visualize the relevance of using even high-order PCs.*

Comment: Fig 3. Details should be provided about why these specific factors (vapor pressure and max temp) were chosen to highlight. All axis labels and legend key items should be larger font; and a heavy horizontal bar separating the two panels would be useful.

*Response: We have provided a reasoning for using these variables in the figure title. We have made legend size larger, and added a solid line to separate the two main panels with distinct environmental variables. Lines 281–286.*

*All changes suggested for the figure have been done.*

Comment: Fig 4. Generating mapped measures of the prediction uncertainty in these suitability gradients would greatly strengthen this study's findings.

https://pmc.ncbi.nlm.nih.gov/articles/PMC3223803/

*Response: We agree. The gradients shown in the picture represent a consensus across models with slightly different parameter settings. On top of that, we are showing where predictions are uncertain because the conditions in such areas are outside model training ranges. We have modified the legend in the figure as well as the figure title to make this fact more evident when interpreting these results. Lines 301–304.*

Comment: pg 4. "vector" (as a verb) should be replaced by "transmit."

*Response: Done.*

------------------------------------------------------

**Reviewer #2:**

Comment: … However, substantial concerns regarding clarity, methodological rationale, model construction, and statistical inference currently limit the interpretability and reliability of the results. In particular, the handling of climate data, use of PCA, model selection strategy, and post-modeling analyses require significant clarification and revision before the manuscript can be adequately evaluated.

*Response: Thank you for your comments and suggestions. We have made significant additions and revisions in our text to clarify our methods section.*

Comment: The final paragraph of the Introduction presents what appear to be results and conclusions, rather than clearly stated objectives or hypotheses.

*Response: We have removed the sentences that seemed like results and conclusions.*

Comment: Study Duration and Internal Inconsistencies

The manuscript refers to a “two-year (2020–2022) field program,” which is internally inconsistent, as that time span covers three calendar years. Based on Ng’eno et al., sampling appears to have occurred from August 2020 to September 2022. The authors should clarify exact start and end months and whether the study should be described as a two-year or three-year dataset. Later references to “three years of analysis” further compound this inconsistency and must be reconciled.

*Response: We have made changes as suggested. See lines 51–53.*

Comment: Methods… This section with vague expository phrasing (“The analyses presented herein”) and refers to “four main steps” that include data preparation, which is not itself an analysis. This weakens clarity and structure. The subheading “Presence–absence data” reads as a sampling methods description and should be reframed as such. The subheading “Environmental data” is misleading, as the section exclusively discusses climatic variables. A more precise label should be used.

*Response: We respectfully disagree. The analyses were developed via a series of methodological steps. This phrasing does not imply that data preparation is an analysis, but rather that it is one step in an analysis. We did change Environmental Data to Climate Data.*

Comment: The exclusion of snow water equivalent is described ambiguously. It is unclear whether sampling occurred during winter months when snow could have been present. I don’t know what is meant by “considerable confusion in results” (e.g., model non-convergence, unstable coefficients). I also do not know what is meant by “extrapolative” in this context, which requires proper explanation and citation.

*Response: To avoid confusion we have excluded the mention of snow water equivalent from our methods. We explored whether using this variable will be important for this study but none of those explorations was presented in this manuscript, therefore, excluding its mention should not represent any issues.*

Comment: The 1 km spatial resolution requires justification given that tick questing occurs at meter-scale.

*Response: We have included considerably more detail justifying the use of variables at this resolution. Lines 110-113.*

Comment: The 8-day temporal aggregation requires justification and ecological grounding.

*Response: We have added a reasoning for the aggregation in our text. Lines 115-116.*

Comment: If only 2020–2022 tick data were used, the ecological rationale for generating climate layers from 1980 must be explicitly stated.

*Response: The data generated before 2020 was not used in this study. Therefore, we have excluded that part from the text.*

Comment: The phrase “variable processing analyses” is unclear and needs precise definition.

*Response: We have listed steps in variable processing to clarify the analyses performed. Lines 119–121.*

Comment: The PCA is described as spanning three years, yet environmental data include months outside the tick dataset (Jan–Jul 2020 and Oct–Dec 2022), which may influence eigenstructure.

*Response: The temporal extent of the PCA is now clarified. Lines 126-128.*

Comments: All six PC axes were retained as predictors. This undermines the stated goal of dimensionality reduction and substantially increases risk of overfitting, data dredging, and loss of interpretability. Typically, only the leading 2–3 axes should be used unless strong justification is provided. The manuscript provides no PCA results, eigenvalues, variance explained, or ecological interpretation of axes, making the analysis impossible to evaluate.

Why are you using all 6 PC variables. Typically you use the first two or three as you are doing the PCA to reduce dimensionality. By doing this this way. I’m concerned about overfitting, data dredging and lack of interpretability of the results.

*Response: We understand that using high order PCs is not common in ecological niche modeling applications. The reason is that they are usually applied as a tool to reduce dimensionality. In our case, a PCA was used not to reduce dimensions, but to characterize all the variance in new orthogonal (non-collinear) dimensions. As such, all PCs are relevant in our exploration because all of them represent unique aspects of environmental variation that can be of relevance to characterize the phenomenon of interest. This is demonstrated by the results from our data-informed model selection process, which show that selected models tend to select most of the PCs, even the ones of high-order. We have included a brief mention of this in our manuscript. Lines 164-168, 260–262.*

Comment: The introduction of niche signal testing would benefit from clearer technical phrasing (e.g., defining niche signal as non-random environmental distribution). The discussion of Cobos and Peterson (2022) unnecessarily emphasizes methodological comparisons rather than clearly stating, which test was used, why it was chosen. It is unclear whether univariate or multivariate tests were actually implemented.

*Response: We have modified our text to clarify that full descriptions of the tests were done in a previous contribution (line 139). Full description of results are now described in lines 238-255.*

Comment: Ecological Niche Model Construction. This section contains multiple serious conceptual and technical issues. The description of model calibration as “contrasting with the usual approach” is vague and unclear.

*Response: We respectfully disagree. We state that the methods for modeling in this paper indeed contrast with the usual "ENM" methodologies. And then we go on to elaborate how our methods in this paper are distinct from the normal approaches, and why. We did review and revise the entire section for clarity. Lines 159-202.*

Comment: This sentence does not make sense. “logistic generalized linear models (GLMs),” this is not how you describe this. It is a generalized linear models with a ? binominal/negative binomial” distribution.

*Response: We believe that there is a slight terminology confusion here. We are indeed using generalized linear models with the argument family set as binomial(link = "logit"). This, together with the fact that our dependent variable options are 0 or 1, makes it logistic. We have added a clarification in parentheses to avoid confusion. Lines 169-170.*

Comment: I have no idea what it means to have a model based on contrasts than “versus”

*Response: We have modified our paragraph to be more accurate. Lines 159–168.*

Comment: Where does pseudoabsence data come from? How are you defining this?

*Response: Other studies commonly derive pseudoabsences from a region around where the presence records are. We are not using pseudoabsence data. Since we performed intensive and consistent sampling across the two states during the ~two year period, we were able to derive presence and absence of questing ticks for the species of interest. This is, we know when we sampled and found a questing tick and when we sampled and did not find it. This does not imply absence of the tick in the region, but absence of questing individuals for each species. We describe how presence and absence records were derived in the last paragraph of the section Presence-absence data. Lines 86-95.*

Comment: The manuscript does not clearly report the total number of candidate models evaluated, how many models passed each filtering step, how many models were retained per species. From the supplement, top model counts vary widely (e.g., 5–6 models for some species and 14 for others), indicating substantial model uncertainty.

*Response: The total number of candidate models tested is reported in the first sentence of the second paragraph in the Model development section (Methods, line 179). We have now included information on how many models were retained per species in the first paragraph of the section Models of questing activity in Results (Lines 258–260).*

*We respectfully disagree with the claim that having multiple models selected indicates uncertainty. The fact that multiple models are selected based on our data and that we used them all to later create a consensus represents variability. No model represents reality perfectly, which is why we decide to use all models that meet selection criteria to generate a consensus. This is a common practice in data-driven modeling approaches.*

Comment: The AIC tables are inconsistently formatted, do not consistently report AIC weights, and are not sorted by ΔAIC values. With such high model uncertainty and broad predictor inclusion, ecological inference is not currently supported.

*Response: We have corrected the inconsistent formatting of the tables.*

*We respectfully disagree with the claim that ecological inference is not supported. After rigorous routines of model selection, we found models that met multiple criteria that demonstrate good performance for all the species. Most of our methodological decisions are traditional in correlative ecological modeling, and the innovations implemented make our methods more robust that common applications in the field. As our model selection process is data-driven, we believe ecological inference is supported.*

Comment: Post-Modeling Analyses. The justification for a 3-week moving window, and the generation of monthly averages is unclear. It is ambiguous that these averages represent, predictions of what?

*Response: We have added a justification for the averaging using the 3-week moving window and by month. Lines 205-212.*

Comment: The Results section omits any presentation of the PCA output.Without PCA loading structure, variance explained, ecological meaning of axes, the subsequent modeling cannot be interpreted. Given the unresolved issues surrounding climate data processing, PCA usage, and extreme model uncertainty, providing peer review on the rest of the results is can not happen from me.

*Response: We have included a description of PCA outcomes in the results. Lines 229–235.*

Comment: Several sections would benefit from tightening and condensation to reduce redundancy.

*Response: We have made changes throughout the entire manuscript to improve its readability.*
