# Supplementary material for: Spatiotemporal dynamics of questing activity by four tick species in the central Great Plains
Source: PLoS One. 2026 Sep 1;21(9):e0351160. doi: 10.1371/journal.pone.0351160 (PMC13533356; doi:10.1371/journal.pone.0351160)
Supplement: Peer review history

# Peer review: Decision Letter 0

**DOI:** 10.1371/journal.pone.0351160.r001

**Authors:** Catherine A. Brissette

10 Dec 2025

Dear Dr. Peterson,

Thank you for submitting your manuscript to PLOS ONE. After careful consideration, we feel that it has merit but does not fully meet PLOS ONE’s publication criteria as it currently stands. Therefore, we invite you to submit a revised version of the manuscript that addresses the points raised during the review process.

Please focus on reviewer's concerns regarding methodological rationale, model construction, and statistical inference.

Please submit your revised manuscript by Jan 24 2026 11:59PM. If you will need more time than this to complete your revisions, please reply to this message or contact the journal office at plosone@plos.org. When you're ready to submit your revision, log on to https://www.editorialmanager.com/pone/ and select the 'Submissions Needing Revision' folder to locate your manuscript file.

- A rebuttal letter that responds to each point raised by the academic editor and reviewer(s). You should upload this letter as a separate file labeled 'Response to Reviewers'.
- A marked-up copy of your manuscript that highlights changes made to the original version. You should upload this as a separate file labeled 'Revised Manuscript with Track Changes'.
- An unmarked version of your revised paper without tracked changes. You should upload this as a separate file labeled 'Manuscript'.

If you would like to make changes to your financial disclosure, please include your updated statement in your cover letter. Guidelines for resubmitting your figure files are available below the reviewer comments at the end of this letter.

If applicable, we recommend that you deposit your laboratory protocols in protocols.io to enhance the reproducibility of your results. Protocols.io assigns your protocol its own identifier (DOI) so that it can be cited independently in the future. For instructions see: https://journals.plos.org/plosone/s/submission-guidelines#loc-laboratory-protocols. Additionally, PLOS ONE offers an option for publishing peer-reviewed Lab Protocol articles, which describe protocols hosted on protocols.io. Read more information on sharing protocols at https://plos.org/protocols?utm_medium=editorial-email&utm_source=authorletters&utm_campaign=protocols.

We look forward to receiving your revised manuscript.

Kind regards,

Catherine A. Brissette, Ph.D.

Academic Editor

PLOS One

Journal Requirements:

When submitting your revision, we need you to address these additional requirements.

1. Please ensure that your manuscript meets PLOS ONE's style requirements, including those for file naming. The PLOS ONE style templates can be found at

https://journals.plos.org/plosone/s/file?id=wjVg/PLOSOne_formatting_sample_main_body.pdf and

https://journals.plos.org/plosone/s/file?id=ba62/PLOSOne_formatting_sample_title_authors_affiliations.pdf

2. Please update your submission to use the PLOS LaTeX template. The template and more information on our requirements for LaTeX submissions can be found at http://journals.plos.org/plosone/s/latex.

3. Thank you for stating the following financial disclosure:

“This material is based upon work supported by the National Science Foundation under Grant No. OIA-1920946.”

Please state what role the funders took in the study.  If the funders had no role, please state: "The funders had no role in study design, data collection and analysis, decision to publish, or preparation of the manuscript."

If this statement is not correct you must amend it as needed.

Please include this amended Role of Funder statement in your cover letter; we will change the online submission form on your behalf.

4. We note that Figures 4 and S5 - S8 in your submission contain map images which may be copyrighted. All PLOS content is published under the Creative Commons Attribution License (CC BY 4.0), which means that the manuscript, images, and Supporting Information files will be freely available online, and any third party is permitted to access, download, copy, distribute, and use these materials in any way, even commercially, with proper attribution. For these reasons, we cannot publish previously copyrighted maps or satellite images created using proprietary data, such as Google software (Google Maps, Street View, and Earth). For more information, see our copyright guidelines: http://journals.plos.org/plosone/s/licenses-and-copyright.

We require you to either (1) present written permission from the copyright holder to publish these figures specifically under the CC BY 4.0 license, or (2) remove the figures from your submission:

1. You may seek permission from the original copyright holder of Figure(s) [#] to publish the content specifically under the CC BY 4.0 license.

We recommend that you contact the original copyright holder with the Content Permission Form (http://journals.plos.org/plosone/s/file?id=7c09/content-permission-form.pdf) and the following text:

“I request permission for the open-access journal PLOS ONE to publish XXX under the Creative Commons Attribution License (CCAL) CC BY 4.0 (http://creativecommons.org/licenses/by/4.0/). Please be aware that this license allows unrestricted use and distribution, even commercially, by third parties. Please reply and provide explicit written permission to publish XXX under a CC BY license and complete the attached form.”

Please upload the completed Content Permission Form or other proof of granted permissions as an "Other" file with your submission.

In the figure caption of the copyrighted figure, please include the following text: “Reprinted from [ref] under a CC BY license, with permission from [name of publisher], original copyright [original copyright year].”

2. If you are unable to obtain permission from the original copyright holder to publish these figures under the CC BY 4.0 license or if the copyright holder’s requirements are incompatible with the CC BY 4.0 license, please either i) remove the figure or ii) supply a replacement figure that complies with the CC BY 4.0 license. Please check copyright information on all replacement figures and update the figure caption with source information. If applicable, please specify in the figure caption text when a figure is similar but not identical to the original image and is therefore for illustrative purposes only.

The following resources for replacing copyrighted map figures may be helpful:

USGS National Map Viewer (public domain): http://viewer.nationalmap.gov/viewer/

The Gateway to Astronaut Photography of Earth (public domain): http://eol.jsc.nasa.gov/sseop/clickmap/

Maps at the CIA (public domain): https://www.cia.gov/library/publications/the-world-factbook/index.html and https://www.cia.gov/library/publications/cia-maps-publications/index.html

NASA Earth Observatory (public domain): http://earthobservatory.nasa.gov/

Landsat: http://landsat.visibleearth.nasa.gov/

USGS EROS (Earth Resources Observatory and Science (EROS) Center) (public domain): http://eros.usgs.gov/#

Natural Earth (public domain): http://www.naturalearthdata.com/

5. If the reviewer comments include a recommendation to cite specific previously published works, please review and evaluate these publications to determine whether they are relevant and should be cited. There is no requirement to cite these works unless the editor has indicated otherwise.

[Note: HTML markup is below. Please do not edit.]

Reviewers' comments:

Reviewer's Responses to Questions

**Comments to the Author**

1. Is the manuscript technically sound, and do the data support the conclusions?

Reviewer #1: Yes

Reviewer #2: No

**********

2. Has the statistical analysis been performed appropriately and rigorously?

Reviewer #1: Yes

Reviewer #2: No

**********

3. Have the authors made all data underlying the findings in their manuscript fully available?

The PLOS Data policy

Reviewer #1: Yes

Reviewer #2: Yes

**********

4. Is the manuscript presented in an intelligible fashion and written in standard English?

Reviewer #1: Yes

Reviewer #2: No

**********

Reviewer #1: In this manuscript, Cobos and coworkers use high-resolution field sampling to develop new methods for ecological niche models that incorporate temporal variation to existing spatial frameworks. These methods are applied to quantify differences in spatio-temporal abundance patterns for four medically-important tick species across a wide swath of OK and KS. The methods are sound and generally well-reported; however, there are several areas that require greater explication.

Note: line number should be added throughout.

***** Major required changes

General: this manuscript should be restructured to be explicitly either i,) a methods paper with a practical example, or ii.) practical medical entomology utilizing a novel method. It's currently a hybrid of the two, greatly complicating the scientific "story."

Pg 6. Justifications/references for the choice of these environmental parameters (esp. srad and wvap) should be included.

Pg 6. Brief mention should be made of (presumed) cluster usage, and general run times for these models to support potential replications.

Pg. 9 "We created a weighed average of all best...." This appears to be an ensemble model; the aggregation choices should be detailed and justified.

Pg. 10. "Post-modeling analyses" The choice of smoother windows here very likely has a major impact on model outputs. Sensitivity analyses should be reported.

Pg 12. First paragraph. This is the first mention of specific PCs. The complex interpretability of these components needs to be made explicit. Secondly, the utility of higher-order PCs beyond 3-4 is very unusual and should be commented on. Lastly, the nature of the extrapolation regions needs further detail (which parameters were outside the raster datasets?).

Pg 14-15. The dynamics displayed in Fig 4 are a core part of this manuscript, yet the discussion as written doesn't really engage with the underlying drivers of presence/true-absence. Wherever possible this section should be reconsidered to incorporate references to bio-ecology beyond "warm-season" vs "cold-season" ticks.

**** Recommended changes

Pg. 9 "Once all candidate models...." A diagram showing the model-selection processes would be an important addition.

Pg 10. Figure 2. This figure has limited utility and could be moved to supplemental utility.

Fig 3. Details should be provided about why these specific factors (vapor pressure and max temp) were chosen to highlight. All axis labels and legend key items should be larger font; and a heavy horizontal bar separating the two panels would be useful.

Fig 4. Generating mapped measures of the prediction uncertainty in these suitability gradients would greatly strengthen this study's findings.

https://pmc.ncbi.nlm.nih.gov/articles/PMC3223803/

**** Minor editorial changes

pg 4. "vector" (as a verb) should be replaced by "transmit."

Reviewer #2: This manuscript addresses an important topic by attempting to characterize tick phenology and environmental niche dynamics across the central Great Plains using multi-year field and climate data. The general framework is promising, and the large spatial scope of environmental data has the potential to yield insight into seasonal patterns of tick activity. However, substantial concerns regarding clarity, methodological rationale, model construction, and statistical inference currently limit the interpretability and reliability of the results. In particular, the handling of climate data, use of PCA, model selection strategy, and post-modeling analyses require significant clarification and revision before the manuscript can be adequately evaluated.

Major Concerns

The final paragraph of the Introduction presents what appear to be results and conclusions, rather than clearly stated objectives or hypotheses.

Study Duration and Internal Inconsistencies

The manuscript refers to a “two-year (2020–2022) field program,” which is internally inconsistent, as that time span covers three calendar years. Based on Ng’eno et al., sampling appears to have occurred from August 2020 to September 2022. The authors should clarify exact start and end months and whether the study should be described as a two-year or three-year dataset. Later references to “three years of analysis” further compound this inconsistency and must be reconciled.

Methods

This section with vague expository phrasing (“The analyses presented herein”) and refers to “four main steps” that include data preparation, which is not itself an analysis. This weakens clarity and structure. The subheading “Presence–absence data” reads as a sampling methods description and should be reframed as such. The subheading “Environmental data” is misleading, as the section exclusively discusses climatic variables. A more precise label should be used.

The exclusion of snow water equivalent is described ambiguously. It is unclear whether sampling occurred during winter months when snow could have been present. I don’t know what is meant by “considerable confusion in results” (e.g., model non-convergence, unstable coefficients). I also do not know what is meant by “extrapolative” in this context, which requires proper explanation and citation.

The 1 km spatial resolution requires justification given that tick questing occurs at meter-scale.

The 8-day temporal aggregation requires justification and ecological grounding.

If only 2020–2022 tick data were used, the ecological rationale for generating climate layers from 1980 must be explicitly stated.

The phrase “variable processing analyses” is unclear and needs precise definition.

The PCA is described as spanning three years, yet environmental data include months outside the tick dataset (Jan–Jul 2020 and Oct–Dec 2022), which may influence eigenstructure.

All six PC axes were retained as predictors. This undermines the stated goal of dimensionality reduction and substantially increases risk of overfitting, data dredging, and loss of interpretability. Typically, only the leading 2–3 axes should be used unless strong justification is provided. The manuscript provides no PCA results, eigenvalues, variance explained, or ecological interpretation of axes, making the analysis impossible to evaluate.

The introduction of niche signal testing would benefit from clearer technical phrasing (e.g., defining niche signal as non-random environmental distribution). The discussion of Cobos and Peterson (2022) unnecessarily emphasizes methodological comparisons rather than clearly stating, which test was used, why it was chosen. It is unclear whether univariate or multivariate tests were actually implemented.

Ecological Niche Model Construction

This section contains multiple serious conceptual and technical issues. The description of model calibration as “contrasting with the usual approach” is vague and unclear. “Logistic generalized linear models” are incorrectly described; the distribution (e.g., binomial) must be stated. The phrase “models based on contrasts versus…” is undefined.

“Ecological niche model calibration was done via methods that contrast rather markedly

with the usual approach to such questions” This sentence does not make sense.

“logistic generalized linear models (GLMs),” this is not how you describe this. It is a generalized linear models with a ? binominal/negative binomial” distribution.

I have no idea what it means to have a model based on contrasts than “versus”

Where does pseudoabsence data come from? How are you defining this?

Why are you using all 6 PC variables. Typically you use the first two or three as you are doing the PCA to reduce dimensionality. By doing this this way. I’m concerned about overfitting, data dredging and lack of interpretability of the results.

The manuscript does not clearly report the total number of candidate models evaluated, how many models passed each filtering step, how many models were retained per species.

From the supplement, top model counts vary widely (e.g., 5–6 models for some species and 14 for others), indicating substantial model uncertainty.

The AIC tables are inconsistently formatted, do not consistently report AIC weights, and are not sorted by ΔAIC values. With such high model uncertainty and broad predictor inclusion, ecological inference is not currently supported.

Post-Modeling Analyses

The justification for a 3-week moving window, and the generation of monthly averages

is unclear. It is ambiguous that these averages represent, predictions of what?

Results Interpretation

The Results section omits any presentation of the PCA output.Without PCA loading structure, variance explained, ecological meaning of axes, the subsequent modeling cannot be interpreted. Given the unresolved issues surrounding climate data processing, PCA usage, and extreme model uncertainty, providing peer review on the rest of the results is can not happen from me.

Minor and Editorial Issues

Several sections would benefit from tightening and condensation to reduce redundancy

**********

what does this mean?). If published, this will include your full peer review and any attached files.

If you choose “no”, your identity will remain anonymous but your review may still be made public.

**Do you want your identity to be public for this peer review?**  For information about this choice, including consent withdrawal, please see our Privacy Policy

Reviewer #1: No

Reviewer #2: **Yes:** Samniqueka Halsey

**********

[NOTE: If reviewer comments were submitted as an attachment file, they will be attached to this email and accessible via the submission site. Please log into your account, locate the manuscript record, and check for the action link "View Attachments". If this link does not appear, there are no attachment files.]

To ensure your figures meet our technical requirements, please review our figure guidelines: https://journals.plos.org/plosone/s/figures

You may also use PLOS’s free figure tool, NAAS, to help you prepare publication quality figures: https://journals.plos.org/plosone/s/figures#loc-tools-for-figure-preparation.

NAAS will assess whether your figures meet our technical requirements by comparing each figure against our figure specifications.

# Author comment: Author response to Decision Letter 1

**DOI:** 10.1371/journal.pone.0351160.r002

20 May 2026

Please see detailed Response to Reviewers document, which was uploaded as a document and is well formatted and more readable than if I were to paste it here.

Attachment Submitted filename: response_reviews.docx

# Editor report: Decision Letter 1

**DOI:** 10.1371/journal.pone.0351160.r003

**Authors:** Catherine A. Brissette

24 May 2026

Spatiotemporal dynamics of questing activity by four tick species in the central Great Plains

PONE-D-25-22057R1

Dear Dr. Peterson,

We’re pleased to inform you that your manuscript has been judged scientifically suitable for publication and will be formally accepted for publication once it meets all outstanding technical requirements.

Within one week, you’ll receive an e-mail detailing the required amendments. When these have been addressed, you’ll receive a formal acceptance letter and your manuscript will be scheduled for publication.

An invoice will be generated when your article is formally accepted. Please note, if your institution has a publishing partnership with PLOS and your article meets the relevant criteria, all or part of your publication costs will be covered. Please make sure your user information is up-to-date by logging into Editorial Manager at Editorial Manager® and clicking the ‘Update My Information' link at the top of the page. For questions related to billing, please contact billing support.

If your institution or institutions have a press office, please notify them about your upcoming paper to help maximize its impact. If they’ll be preparing press materials, please inform our press team as soon as possible -- no later than 48 hours after receiving the formal acceptance. Your manuscript will remain under strict press embargo until 2 pm Eastern Time on the date of publication. For more information, please contact onepress@plos.org.

Kind regards,

Catherine A. Brissette, Ph.D.

Academic Editor

PLOS One

Additional Editor Comments (optional):

Reviewers' comments:

# Editor report: Acceptance letter

**DOI:** 10.1371/journal.pone.0351160.r004

**Authors:** Catherine A. Brissette

PONE-D-25-22057R1

PLOS One

Dear Dr. Peterson,

I'm pleased to inform you that your manuscript has been deemed suitable for publication in PLOS One. Congratulations! Your manuscript is now being handed over to our production team.

At this stage, our production department will prepare your paper for publication. This includes ensuring the following:

* All references, tables, and figures are properly cited

* All relevant supporting information is included in the manuscript submission,

* There are no issues that prevent the paper from being properly typeset

You will receive further instructions from the production team, including instructions on how to review your proof when it is ready. Please keep in mind that we are working through a large volume of accepted articles, so please give us a few days to review your paper and let you know the next and final steps.

Lastly, if your institution or institutions have a press office, please let them know about your upcoming paper now to help maximize its impact. If they'll be preparing press materials, please inform our press team within the next 48 hours. Your manuscript will remain under strict press embargo until 2 pm Eastern Time on the date of publication. For more information, please contact onepress@plos.org.

You will receive an invoice from PLOS for your publication fee after your manuscript has reached the completed accept phase. If you receive an email requesting payment before acceptance or for any other service, this may be a phishing scheme. Learn how to identify phishing emails and protect your accounts at https://explore.plos.org/phishing.

If we can help with anything else, please email us at customercare@plos.org.

Thank you for submitting your work to PLOS One and supporting open access.

Kind regards,

PLOS ONE Editorial Office Staff

on behalf of

Dr. Catherine A. Brissette

Academic Editor

PLOS One
